# The effectiveness of an extra-curricular lecture for STI prevention and sexual education

**DOI:** 10.1017/S0950268823000079

**Published:** 2023-02-10

**Authors:** M. Reinholz, T. Nellessen, E. Wei, S. Zippel, C. Fuchs, T. Kaemmerer, B. M. Clanner-Engelshofen, L. H. Frommherz, M. Rummel, L. E. French, P.-C. Stadler

**Affiliations:** 1 Department of Dermatology and Allergy, University Hospital, LMU Munich, Frauenlobstr. 9-11, 80337 Munich, Germany; 2 Dr. Phillip Frost Department of Dermatology & Cutaneous Surgery, Miller School of Medicine, University of Miami, Miami, 33136, Florida, USA

**Keywords:** Chlamydia, gonorrhoea, sexually transmitted infections, vaccination (immunisation), vaccine preventable diseases

## Abstract

While the incidence of infections with the human immunodeficiency virus largely remained unchanged in Germany, an increase of other sexually transmitted infections (STIs) was observed. The aim was to analyse the effectiveness of our sexual education lecture for students in improving the awareness, knowledge and prevention of STIs. We conducted a cross-sectional survey after students had attended our extra-curricular lecture at the Department of Dermatology of the Ludwig-Maximilians-University of Munich, Germany (LMU). We compared the data with a previously performed study in which the same survey was carried out before the lecture had started. A total of 5866 questionnaires were included in the analysis. After attending the lecture significantly more students were aware of STIs (syphilis: 36.8% (before) *vs.* 63.5% (after); chlamydia: 30.5% *vs.* 49.3%; gonorrhoea: 22.4% *vs.* 38.2%; human papillomaviruses (HPV): 17.7% *vs.* 30.2%), the transmission pathways of STIs (oral: 36.6% *vs.* 82.6%; vaginal: 81.8% *vs.* 97.3%; anal: 42.8% *vs.* 94.0%; penile: 68.7% *vs.* 92.1%), knew that the HPV vaccination is directed against a virus (36.8% *vs.* 56.9%) and were interested in receiving a vaccination (57.7% *vs.* 78.8%). This study demonstrates the positive educative effects of our lecture for awareness and improved knowledge of STIs. To satisfy the need for a comprehensive sexual education, a combination of school and health facility-based programmes should be implemented as one single lecture cannot convey the entire information about STIs.

## Introduction

The incidence of bacterial sexually transmitted infections (STIs), as well as the prevalence of drug resistance has strikingly increased in the last decade, thus resulting in complex treatment regimens for STIs and a significant burden for the population and health-care systems [1]. In Germany, infections with *Treponema pallidum*, causing syphilis – a disease that can affect the central nervous system and potentially lead to death in later stages – increased from 6834 cases to 7889 between 2015 and 2019 [2, 3]. In contrast, the incidence of infections with the human immunodeficiency virus (HIV) [4] has largely remained unchanged in Germany over the last 10 years, while it remains challenging in developing countries [2, 5]. In 2019, the worldwide prevalence of HIV was about 38 million people [6], and Germany is one of the countries with the lowest HIV infection incidence in Europe, most likely due to its successful preventive work [7]. In addition to the increased antibiotic resistance, one explanation for the rising incidence of STIs other than HIV is higher risk sexual behaviour, especially common in men who have sex with men (MSM), since HIV became treatable and pre-exposure prophylaxis regime became available [8]. In addition to infections with *T. pallidum*, a similar rising trend is seen for other bacterial STIs, including infections with *Chlamydia trachomatis* or *Neisseria gonorrhoeae*, which might be facilitated due to their mostly asymptomatic course [9]. Chlamydia belongs to the most common STIs in Germany, with a prevalence of up to 5% in young adults. It can cause, like gonorrhoea, a chronic pelvic inflammatory disease with infertility in the long term. Chlamydia is currently the main cause of infertility in young women [10]. Furthermore, some *N. gonorrhoeae* strains have developed a resistance to most commonly used antibiotics in the last decades, thus leaving ceftriaxone ± azithromycin the last remaining effective antibiotic regime for routine use [11]. In addition, a multidrug-resistant strain from Southeast Asia was newly discovered; outlining the relevance of sex tourism and travel diseases [1, 12]. Other STIs include infections with human papillomaviruses (HPV). The high-risk HPV subtypes 16 and 18 can cause a significant long-term health impact as they are associated with cancer development in different regions – anus, vagina, vulva, penis and oropharynx – and associated with 70% of cervix carcinoma [13, 14]. Although the vaccination against HPV offers the best possible protection against an infection with oncogenic HPV subtypes, only 31.3% are vaccinated against HPV at the age of 15 in Germany [15]. This shows that while the awareness for HIV is good in Germany [16], additional focus on prevention should be on other STIs.

## Methods

From March 2019 to June 2019 (cohort 2), we conducted a cross-sectional anonymous survey using a questionnaire evaluating the knowledge of students about transmission and prevention of STIs after they had attended a 3 h extra-curricular lecture focusing on STIs at the Department of Dermatology of the Ludwig-Maximilians-University of Munich, Germany (LMU). The participating students were between the 8 and 10 school year from different schools and participation at the lecture took place after registration by the class teachers. In one lecture, around 80 students attended and the lecture was being held at the Department of Dermatology daily on weekdays. The students who participated in the survey gave their consent before the paper questionnaire was distributed. The data were compared with a previously performed study in which the same survey was carried out before the lecture had started (cohort 1) [17]. In order to analyse the differences of knowledge before and after the lecture, the same procedure was carried out for different student cohorts to ensure that the students had no preknowlegde about the questionnaire. The recruitment for cohort 1 took place between September 2018 and February 2019. The objective of the present study was to determine the difference in awareness and level of knowledge of STIs between cohort 1 and cohort 2 in order to analyse the effectiveness of an extra-curricular lecture in improving the awareness and knowledge of STIs.

### Statistical analyses

Metric variables were reported as mean values ± standard deviation (s.d.). Reported percentages refer to the applicable cases. Group comparisons were made using the *χ*^2^ tests. A *P*-value of 0.05 or less was considered significant (*P* *** ≤ 0.001, *P* ** ≤ 0.01, *P* * ≤ 0.05).

### Questionnaire design

In a multiple-choice question format, both cohorts were asked about their knowledge and awareness of STIs with the same questionnaire. The questionnaire consisted of 27 questions, five questions related to socio-demographics, and the remaining 22 to general knowledge about STIs, their modes of transmission and possible prevention and protection measures. We investigated the awareness of infections with *C. trachomatis*, *N. gonorrhoeae*, syphilis, *HPV*, *HIV*, as well as diseases such as genital herpes, genital warts, scabies and syphilis. We assumed that students were unaware of the association between genital warts and HPV and therefore studied them as separate entities [18]. We further investigated school students’ knowledge of STI transmission, HPV vaccination, vaccination status as well as the interest in receiving a HPV vaccination and the reasons for not getting vaccinated. The questions were carried out in a single and multiple-choice format.

## Results

### Socio-demographics

A total of 5866 questionnaires were included in the study. In cohort 1, the final study population was 3834, of which 47.9% were male, 50.4% were female and 1.3% were non-binary with a mean age of 15.26 years. In cohort 2, the final study population was 2032, of which 50.9% were male, 48.5% female and 0.6% were non-binary with a mean age of 15.84 years. Furthermore, 25.7% of cohort 1 and 34.3% of cohort 2 attended one or both youth examinations (Table 1). Youth examinations are preventive examinations for teenagers in Germany which should be performed twice. The first one between age 12 and 14 and the second one between the age of 16 and 17. Those screenings are performed by paediatricians and include educating the teenagers on issues related to sexuality and contraceptions including protections against STIs.

**Table 1.** Demographic data of the study population regarding gender, school type, age distribution (mean age and age span) and youth examinations

	Cohort 1		Cohort 2	
	*n* = 3834		*n* = 2032	
	*n*	%	*n*	%
Gender				
Male	1836	47.9	1034	50.9
Female	1931	50.4	985	48.5
Non-binary	49	1.3	13	0.6
No gender information	18	0.5	None	None
School type				
Grammar school	980	25.6	948	46.7
Secondary school	816	21.3	1015	49.9
Other school types	2038	53.2	69	3.4
Mean age	15.26	s.d. ± 0.856	15.84	s.d. ± 0.706
Youth examinations	987	25.7	700	34.4

### Knowledge about STIs

**Fig. 1.**
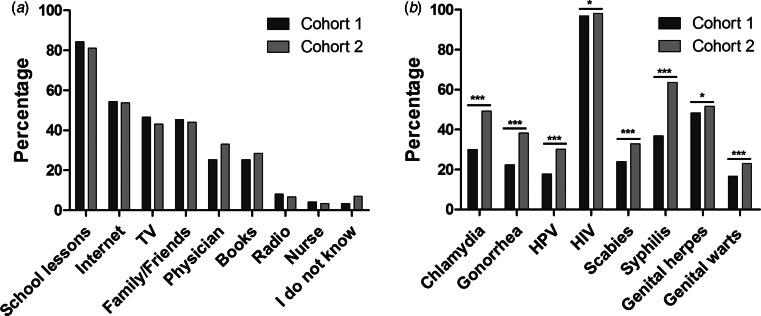
(a) Acquirement of knowledge about the different STIs in comparison between cohort 1 (*n* = 3755) and cohort 2 (*n* = 2006). Cohort 1 *vs.* 2: school lessons (84.3% *vs.* 81.0%), internet (54.4% *vs.* 53.7%), TV (46.5% *vs.* 43.0%), family/friends (45.4% *vs.* 44.0%), physician (25.3% *vs.* 33.0%), books (25.3% *vs.* 28.4%), radio (8.0% *vs.* 6.6%), nurse (4.1% *vs.* 3.3%), I do not know (3.2% *vs.* 6.9%). (b) Awareness of the different STIs before (cohort 1, *n* = 3755) and after the lecture (cohort 2, *n* = 2006). Cohort 1 *vs.* 2: chlamydia (30.5% *vs.* 49.3%; *P* < 0.001), gonorrhoea (22.4% *vs.* 38.2%; *P* < 0.001), HPV (17.7% *vs.* 30.2%; *P* < 0.001), HIV (96.9% *vs.* 98.1%; *P* = 0.011), scabies (23.9% *vs.* 32.9%; *P* < 0.001), syphilis (36.8% *vs.* 63.5%; *P* < 0.001), genital herpes (48.4% *vs.* 51.7%; *P* = 0.017), genital wards (16.7% *vs.* 23%; *P* < 0.001).

In cohort 1, the most common source of knowledge acquisition about STIs was school lessons with 84.3%, followed by the internet with 54.4%, television with 46.5%, family/friends with 45.4%, physicians with 25.3% and books with 25.3%. In cohort 2, a similar rank order was found: 81.0% stated school lessons, 53.7% internet, 44.0% family/friends, 43.0% television, 33.0% physicians and 28.4% books (Fig. 1a).

Before the lecture, 96.9% had heard about HIV, 48.4% about genital herpes, 36.8% about syphilis, 30.5% had heard about chlamydia, 23.9% about scabies, 22.4% about gonorrhoea, 17.7% about HPV and 16.7% about genital warts. After the lecture, 98.1% had heard about HIV, 63.5% about syphilis, 51.7% about genital herpes, 49.3% had heard about chlamydia, 38.2% about gonorrhoea, 32.9% about scabies, 30.2% about HPV and 23.0% about genital warts (Fig. 1b). The students’ awareness after the lecture was significantly higher for all named STIs (*P* < 0.001; Fig. 1b). There was no difference seen between males and females (Table 2).

**Table 2.** Knowledge about different sexually transmitted infections (STIs)

	Cohort 1		Cohort 2		
	*n* = 3755 *n*_male_ = 1786 *n*_female_ = 1906		*n* = 2006 *n*_male_ = 1019 *n*_female_ = 974		
Heard about	*n*	%	*n*	%	*P*-value
Chlamydia					
Overall	1146	30.5	989	49.3	<0.001
Male	398	22.3	439	43.1	<0.001
Female	727	38.1	539	55.3	<0.001
Gonorrhoea					
Overall	841	22.4	767	38.2	<0.001
Male	423	23.7	403	39.5	<0.001
Female	403	21.1	353	36.2	<0.001
HPV					
Overall	663	17.7	606	30.2	<0.001
Male	181	10.1	234	23.0	<0.001
Female	467	24.5	363	37.3	<0.001
HIV					
Overall	3639	96.9	1967	98.1	0.011
Male	1736	97.2	994	97.5	0.585
Female	1842	96.6	960	98.6	<0.001
Scabies					
Overall	896	23.9	660	32.9	<0.001
Male	420	23.5	338	33.2	<0.001
Female	453	23.8	314	32.2	<0.001
Syphilis					
Overall	1383	36.8	1273	63.5	<0.001
Male	714	40.0	644	63.2	<0.001
Female	627	32.9	616	63.2	<0.001
Genital herpes					
Overall	1819	48.4	1038	51.7	0.017
Male	848	47.5	555	54.5	<0.001
Female	934	49.0	473	48.6	0.823
Genital warts					
Overall	628	16.7	462	23.0	<0.001
Male	296	16.6	237	23.3	<0.001
Female	319	16.7	215	22.1	<0.001

**Fig. 2.**
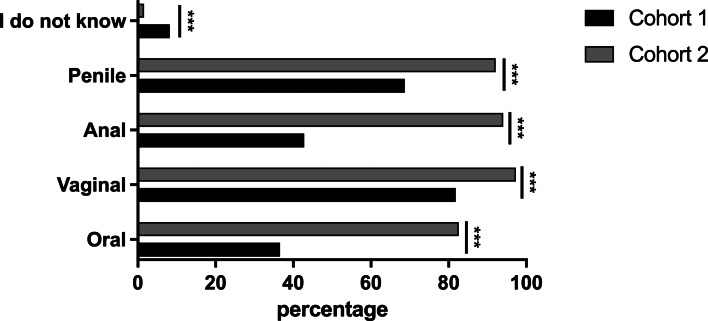
Students’ knowledge about the transmission of STIs in comparison between cohort 1 (*n* = 3755) and cohort 2 (*n* = 2032). Cohort 1 *vs.* 2: oral (36.6% *vs.* 82.6%; *P* < 0.001), vaginal (81.8% *vs.* 97.3%; *P* < 0.001), anal (42.8% *vs.* 94.0%; *P* < 0.001), penile (68.7% *vs.* 92.1%; *P* < 0.001).

Concerning the transmission of STIs, 81.8% of the students in cohort 1 stated that STIs can be transmitted by the vaginal, 68.7% penile, 42.8% anal and 36.6% oral route. In comparison, in cohort 2, 97.3% of the students stated that STIs can be transmitted by the vaginal, 92.1% penile, 94.0% anal and 82.6% oral route. For all transmission pathways, the knowledge was significantly higher after the lecture (*P* < 0.001; Fig. 2).

### HPV vaccination

In cohort 2, significantly more students (59.0%) were aware of HPV vaccination, in contrast to cohort 1 with 36.9% (*P* < 0.001, Table 3a). In cohort 1, only 36.8% of the students did know that HPV vaccination is directed against viruses, whereas significantly more students knew it after the lecture with 56.9% (*P* < 0.001; Table 3b). There were no differences between male or female.

**Table 3.** Questions concerning the HPV vaccination

(a)	Cohort 1		Cohort 2		
Have you heard of the HPV vaccination?	*n* = 3770 *n*_male_ = 1840 *n*_female_ = 1895		*n* = 2012 *n*_male_ = 1021 *n*_female_ = 978		
	*n*	%	*n*	%	*P*-value
Overall	1390	36.9	1188	59.0	<0.001
Male	441	24.0	449	44.0	<0.001
Female	955	50.4	730	74.6	<0.001
	Cohort 1		Cohort 2		
Are you vaccinated against HPV?	*n* = 1372 *n*_male_ = 403 *n*_female_ = 945		*n* = 2025 *n*_male_ = 1028 *n*_female_ = 984		
	*n*	%	*n*	%	*P*-value
Overall	372	27.1	459	22.7	<0.001
Male	78	19.4	92	8.9	<0.001
Female	286	30.3	363	36.9	<0.001
	Cohort 1		Cohort 2		
Are you interested in receiving a HPV vaccination?	*n* = 898 *n*_male_ = 284 *n*_female_ = 598		*n* = 1806 *n*_male_ = 978 *n*_female_ = 818		
	*n*	%	*n*	%	*P*-value
Overall	543	60.5	1421	78.7	<0.001
Male	140	49.3	772	78.9	<0.001
Female	395	66.1	643	78.6	<0.001
	Cohort 1		Cohort 2		
If not, what are the counterarguments	*n* = 355 *n*_male_ = 144 *n*_female_ = 203		*n* = 385 *n*_male_ = 206 *n*_female_ = 175		
	*n*	%	*n*	%	*P*-value
I do not need a vaccination					
Overall	114	32.1	93	24.2	<0.001
Male	57	39.6	72	35.0	0.377
Female	55	27.1	21	12.0	<0.001
I am concerned about possible side effects					
Overall	56	15.8	86	22.3	0.024
Male	6	4.2	29	14.1	0.002
Female	48	23.6	57	32.6	0.053
I do not know enough about the vaccination					
Overall	142	40.0	141	36.6	0.345
Male	55	38.2	63	30.6	0.138
Female	87	42.9	77	44.0	0.823

(b)	Cohort 1			Cohort 2			
What is the role of the HPV vaccination?	*n* = 1266 *n*_male_ = 369 *n*_female_ = 879			*n* = 2013 *n*_male_ = 1024 *n*_female_ = 976			
	*n*		%	*n*		%	*P*-value
Overall	Virus	446	36.8	Virus	1146	56.9	<0.001
	Bacteria	121	9.6	Bacteria	141	7.0	
	Fungi	48	3.8	Fungi	66	3.3	
	I do not know	631	49.8	I do not know	660	32.8	
Male	Virus	127	34.4	Virus	516	50.4	<0.001
	Bacteria	28	7.6	Bacteria	64	6.3	
	Fungi	20	5.4	Fungi	48	4.7	
	I do not know	194	52.6	I do not know	396	38.7	
Female	Virus	327	37.2	Virus	624	63.9	<0.001
	Bacteria	93	10.6	Bacteria	76	7.8	
	Fungi	28	3.2	Fungi	16	1.6	
	I do not know	431	49.0	I do not know	260	26.6	

In cohort 2, significantly fewer students were vaccinated against HPV (22.7%) in contrast to cohort 1 (27.1%; *P* < 0.001). In both cohorts, males were less vaccinated against HPV with an average of 14.2% compared to females with an average of 33.6% (*P* < 0.001; Table 3a).

After the lecture, a significantly higher number of students (78.6%) were interested in getting a HPV vaccination, compared to cohort 1 (60.5%; *P* < 0.001). The increase was higher for male (49.3% *vs.* 78.9%; *P* < 0.001), compared to female (66.1% *vs.* 78.6%; *P* < 0.001) resulting in the fact that before the lecture significantly more females were interested in HPV vaccination compared to males whereas after the lecture it was the same amount. For those who were not interested in getting a HPV vaccination, we asked them for the reasons. After the lecture, significantly fewer students stated that they do not need a vaccination; in cohort 1, 32.1% compared to cohort 2 with 24.2% (*P* < 0.001). But on the other hand, the students were slightly more afraid of side effects of the vaccination after the lecture (cohort 1 15.8% *vs.* cohort 2 22.3%; *P* = 0.024) and up to one-third of students still thought that they do not know enough about the vaccination which did not change significantly after the lecture (40.0% *vs.* 36.6%; *P* = 0.345; Table 3a).

## Discussion

As predicted, a significant higher number of students had heard about the different STIs – including infections with HIV, *C. trachomatis*, *N. gonorrhoeae* and HPV – and their transmission pathways, especially oral and anal transmission, after the lecture (Table 2). For HIV, the awareness was already high (90%) before the lecture, whereas the knowledge about other bacterial and non-bacterial STIs increased significantly after the lecture. Nevertheless, still only 41.3% of the students knew about other STIs except HIV after the lecture and this might be due to a lack of attention or to the large amount of new information provided in a single 3 h lecture. The knowledge was especially low about the quite common and often asymptomatic infections with *C. trachomatis*, *N. gonorrhoeae* and HPV with only 39.2% in cohort 2. These data reflect the importance and need for better sexual education on STIs in young adults as the long-term health effects of those infections can be infertility and carcinoma development [2, 9, 10].

Furthermore, for all students, school lessons were the most important source of information, while physicians with approximately 29.1% played a minor role, showing that the role of physicians and health-care systems in sexual education should be improved.

To increase the awareness of HPV, as well as HPV vaccination rates, different countries already implemented ‘school-based vaccination programmes’. Studies were able to show the benefit of those programmes with a vaccination rate of 75% in a study from Canada [19] and 88% in a study from the USA [20] underlining the importance of school-based vaccination programmes as additional tools to reach a higher vaccination rate. The combination of both school- and health facility-based programmes might have the best effect to increase knowledge as well as vaccination rates for HPV [21]. This could be also shown in our study where a healthy facility-based programme was performed and the interest in receiving a HPV vaccination increased significantly from 57.7% to 78.8%.

While interest in receiving HPV vaccination increased significantly, vaccination rates were very low in both cohorts with an average of 29.4%, and only 8.9% in male in cohort 2. The Robert-Koch-Institute (RKI) suggested that in order to reduce the incidence of HPV-associated carcinomas, a vaccination rate of at least 22.3% for males is needed, provided that 44.6% of females are vaccinated [15]. Especially for MSM, it is important that also males get vaccinated [22, 23]. According to the Federal Center for Health Education (BZgA) in Germany, the most important reason for a vaccination against HPV is the recommendation by a physician, underlining the importance of youth examinations which are performed by paediatricians [24, 25]. Strikingly in our study, physicians only played a minor role in gaining information about STIs, which thereby needs to be improved and might be due to the low percentage of students attending those youth examinations with 25.7% in cohort 1 and 34.4% in cohort 2. Furthermore, Rieck *et al*. already showed that participation in youth examinations is associated with increased HPV vaccination rates, but the participation rate is not high, which reflects the results of our study [26].

One way to increase the participation rate in such youth examinations is to introduce an invitation system for those examinations, which already has been shown to increase the participation rate by 25% [27]. However, in the case of Germany, there is currently no central immunisation registry existing, making it difficult to introduce an invitation or reminder systems for unvaccinated people [25, 28]. Nevertheless, a study by the BZgA showed that 71.0% of the respondents would welcome being reminded by post, SMS or E-mail for the next vaccination appointment [29], underlining the importance of an implementation of such an invitation or reminder systems.

In terms of the strengths and limitations of our study, one of the most important strengths is the large number of participants so that statistically relevant conclusions could be made and the cohorts could be compared as best as possible despite the fact that the cohorts did not consist of the same students, which is the main limitation of the study. This is due to the fact that otherwise the students would have already known the questionnaire in advance and might have been influenced by it when attending the lecture. Despite this, the socio-demographics, which are quite the same in both groups (age and gender), proved that the groups are well comparable.

In conclusion, our study shows that the extra-curricular lecture at the LMU has a substantial effect on the awareness and knowledge of STIs, but one lecture is not sufficient to convey the entire information about STIs, their transmission, prevention and treatment. We suggest an implementation of health facility-based programmes, including such lectures on a regular basis, as well as school-based programmes, such as vaccination programmes. The healthy facility-based programmes should focus more on STIs other than HIV, as the knowledge about HIV in particular is quite high among students. Furthermore, in Germany in particular, the participation rate in youth examinations should be increased, for example, by means of invitation systems. Accordingly, the most important suggestions to be considered are to increase the attendance in the youth examinations, and structural adaptations, such as regular extra-curricular lectures, and school vaccination programmes to increase the vaccination rate and the awareness of STIs.

## Data Availability

The data that support the findings of this study are available from the authors.
